# Acrylamide Intake and Metabolic Syndrome Risk: The Tehran Lipid and Glucose Study

**DOI:** 10.1002/fsn3.70038

**Published:** 2025-04-18

**Authors:** Firoozeh Hosseini‐Esfahani, Azam Ildarabadi, Shahrzad Daei, Glareh Koochakpoor, Amene Nematollahi, Parvin Mirmiran, Fereidoun Azizi

**Affiliations:** ^1^ Nutrition and Endocrine Research Center, Research Institute for Endocrine Sciences Shahid Beheshti University of Medical Sciences Tehran Iran; ^2^ Maragheh University of Medical Sciences Maragheh Iran; ^3^ Department of Food Safety and Hygiene, School of Health Fasa University of Medical Sciences Fasa Iran; ^4^ Endocrine Research Center, Research Institute for Endocrine Sciences Shahid Beheshti University of Medical Sciences Tehran Iran

**Keywords:** abdominal obesity, acrylamide, dysglycemia, dyslipidemia, hypertension, metabolic syndrome

## Abstract

This cohort study aimed to determine the relationship between acrylamide intake and the risk of metabolic syndrome (MetS) and its risk factors in Tehranian adults. A total of 1762 men and 2786 women, respectively, with a mean ± SD age of 38.6 ± 14.3 and 35.9 ± 11.8 years and body mass index of 25.8 ± 4.31 were evaluated in this secondary analysis of the Tehran Lipid and Glucose Study. Dietary data were collected using a standard food frequency questionnaire. Total acrylamide intake was computed using the amount of acrylamide measured in 30 food items. MetS was defined according to the Iranian‐modified National Cholesterol Education Program. Multivariable Cox proportional hazard regression models were used to estimate the incidence of MetS and its risk factors associated with acrylamide intake, considering confounding factors (e.g., age, sex, physical activity, body mass index, smoking). During the average follow‐up of 6.23 ± 2.58 years, 1279 (28% of total) subjects had MetS incidence. The incidence of MetS was not associated with quartiles of acrylamide intake. After adjusting for confounding factors, participants in the fourth quartile of acrylamide intake had 15% and 21% higher risk of high triglyceride and high waist circumference, respectively. Moreover, acrylamide intake was positively related to high blood pressure incidence (Hazard ratio [Confidence interval] Q1‐Q4: 1, 1.0 [0.92–1.08], 1.10 [1.02–1.19], 1.16 [1.07–1.26], *p* trend = 0.003). Based on findings, no apparent association was observed between acrylamide intake and MetS incidence. Long‐term intake of acrylamide is associated with an increased risk of hypertriglyceridemia, high blood pressure, and abdominal obesity.

## Graphical abstract

No apparent association was observed between acrylamide intake and MetS incidence. Positive relationships between acrylamide intake and central obesity were observed after adjusting for multiple confounding factors. Long‐term intake of acrylamide is associated with an increased risk of hypertriglyceridemia and high blood pressure.

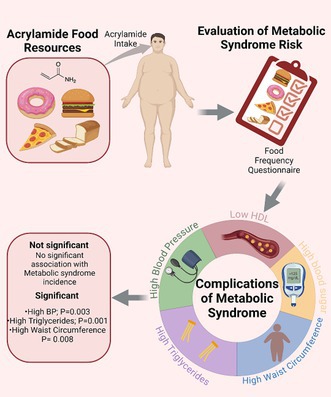

## 1 Introduction

While individuals who smoke cigarettes or work in industrial occupations (e.g., textile manufacturing, oil, water treatment) are commonly exposed to acrylamide (Smith and Oehme 1991; Wang et al. 2020), individuals outside of these situations are regularly exposed through the consumption of starches and nonreducing sugars, where cooking these foods at high temperatures and low moisture leads to the production of acrylamide through the Maillard reaction (Arisseto et al. 2007). The median intake of acrylamide was estimated to be in the wide range of 0.02–1.53 μg/kg body weight per day in 101 studies based on dietary questionnaires across and within populations; however, the level of acrylamide intake, which has adverse effects on human health, is still unknown due to variations of acrylamide in different foods and between the same foods (Timmermann et al. 2021).

The World Health Organization International Cancer Research Center has included acrylamide in the list of possible carcinogenic compounds to humans based on its genotoxicity and carcinogenicity in rodents (Virk‐Baker et al. 2014). In vivo and in vitro rodent models showed that acrylamide leads to insulin resistance and increased blood sugar through oxidative stress on beta cells, disrupting glucose metabolism and insulin signaling pathways. In humans, higher hemoglobin adducts of acrylamide (HbAA) levels were associated with insulin resistance and lower blood insulin in a population‐based survey (Lin et al. 2009; Marković Filipović and Karan 2022). Wang et al. (2020) showed that urinary metabolites of acrylamide were associated with fasting blood glucose (FBG) elevation in a dose‐dependent manner and increased oxidative DNA damage and lipid peroxidation. Also, acrylamide was associated with obesity in mice (Lee and Pyo 2019). The level of hemoglobin adducts of glycidamide (HbGA) was associated with higher obesity, central obesity, and overweight among the US population (Huang et al. 2018). Previous cross‐sectional studies showed that acrylamide disturbs thyroid homeostasis (Lin et al. 2015) and induces insulin resistance (Lin et al. 2009), so acrylamide exposure leads to obesity‐related disorders due to disturbing metabolic homeostasis (Huang et al. 2018). In the US population, HbGA/HbAA had a positive correlation with triglyceride (TG) and a negative correlation with high‐density lipoprotein cholesterol (HDL‐C). Long‐term acrylamide intake induced oxidative stress and chronic inflammation (Cheang et al. 2021).

Considering that the simultaneous appearance of metabolic abnormalities, including hyperglycemia, dyslipidemia, high blood pressure (BP), and central obesity, has been defined as metabolic syndrome (MetS) (Grundy et al. 2004), the question is raised whether acrylamide contributes to the progression of MetS. Answering the question becomes more critical when it has been estimated that more than a quarter of the world's adults suffer from MetS (Ranasinghe et al. 2017), and having MetS can increase the risk of diabetes, cardiovascular disease, and mortality rate (Tune et al. 2017). Few cross‐sectional studies have demonstrated the association between MetS and acrylamide (Hung et al. 2021; Wan et al. 2022). Therefore, this cohort study was conducted to determine the relationship between acrylamide intake and the risk of MetS and its risk factors' incidence in a group of Tehranian adults.

## 2 Materials and Methods

**FIGURE 1:**
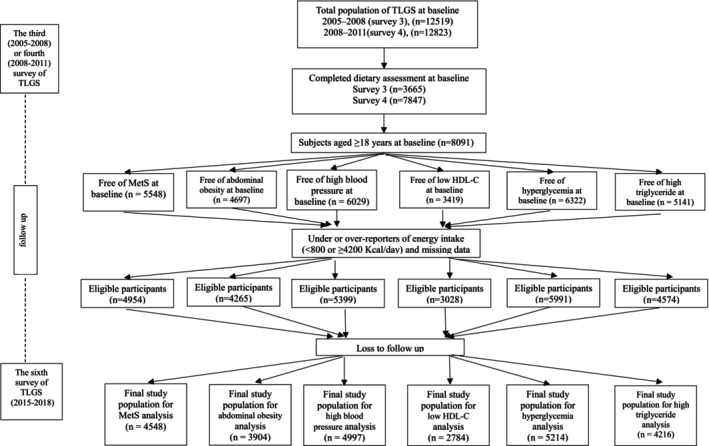
Outline of study participants' selection process for the association of acrylamide intake and risk metabolic syndrome and its risk factors incidence. HDL‐C, High density lipoprotein cholesterol; MetS, Metabolic syndrome.

The Tehran lipid and glucose study (TLGS) was performed prospectively on the 13th district of Tehran (the capital of Iran) residents to determine risk factors for noncommunicable diseases (Azizi et al. 2009; Hosseini‐Esfahani et al. 2018a). The participants of TLGS were selected using the multistage stratified cluster random sampling technique. The first observation study was performed from 1999 to 2001 on 15,005 subjects aged ≥ 3 years. Follow‐up observations were performed every 3 years: 2002–2005 (survey 2), 2005–2008 (survey 3), 2008–2011 (survey 4), 2012–2015 (survey 5), and 2015–2018 (survey 6) to identify the development of disease and other risk factors. For this secondary analysis, of subjects participating in surveys 3 and 4 (baseline of our study), respectively, 3665 and 7847 individuals were randomly selected to complete the dietary assessment (66% of the total population). Of the 8091 subjects aged ≥ 18 years, those with a MetS diagnosis at baseline (Azizi et al. 2010) and pregnant or lactating women were excluded. Also, individuals with over‐ or under‐reporting of energy intake (≥ 4200 or < 800 kcal/day) (*n* = 780) were omitted (Willett 1998). Other independent lines of exclusion were done for MetS risk factors, including abdominal obesity, high BP, low HDL‐C, high FBG, and high TG (Figure 1). A total of 4954 adult women and men with accessible biochemical, anthropometric, and dietary data were selected as the baseline population and were tracked until survey 6. Subjects who did not provide follow‐up data were excluded from participants; as a result, 4548 subjects were included in the final analysis. The study participants' selection process outlines for the association of acrylamide and MetS or its risk factors incidence are shown in Figure 1.

All participants signed a written informed consent form before participating in this study. The study was performed according to the Declaration of Helsinki; the study proposal was approved by the Research Institute for Endocrine Sciences ethics committee, Shahid Beheshti University of Medical Sciences (Tehran, Iran) IR.SBMU.ENDOCRINE.REC.1402.067.

## 3 Dietary Assessment

Trained investigators (high experience in interviewing and filling out the dietary questionnaire) collected dietary data by applying a valid and reliable semiquantitative food frequency questionnaire (FFQ) through face‐to‐face personal interviews (Mirmiran et al. 2010; Esfahani et al. 2010). The FFQ includes 168 items of foods with specific serving sizes, measured in grams. The usual frequency of food item intakes was asked during the last year on a daily, weekly, monthly, or yearly basis. The portion amounts of eaten foods were changed from household measurements to grams per day in such a way that participants' frequency of consumption in each period was converted to days. Then, it was multiplied by the amount of grams per serving.

Due to the incompleteness and limited data on the nutrient content of cooked food items in the Iranian Food Composition Table (FCT), the United States Department of Agriculture (USDA) FCT data were applied to break down the nutrient composition of food items (e.g., bread, legume, nuts, white or red meat). The Iranian FCT was used for national foods that could not be incorporated into the USDA FCT.

After accounting for cooking and food preparation methods, acrylamide was determined for 30 food items during analysis (Nematollahi et al. 2019; Nematollahi et al. 2020a; Nematollahi et al. 2020b).

The acrylamide content of traditional and industrial bread, bakery products (cakes and biscuits), confectionary products (pastries and chocolates), snacks (potato‐based and corn‐based), coffee powder, roasted nuts (almonds, pistachios, hazelnuts, peanuts, and edible seeds), and fast foods (Pizza, hamburger, and sausage) was computed. Food's acrylamide concentration was measured using a dispersive liquid–liquid micro‐extraction system coupled with gas‐chromatography mass spectrometry (Nematollahi et al. 2019; Nematollahi et al. 2020a; Nematollahi et al. 2020b).

The acrylamide and nutrient contents of food items were estimated cumulatively beyond follow‐up surveys from the baseline until the last follow‐up survey or the time that MetS or its risk factors were determined.

## 4 Physical Activity Measurements

An expert interviewer applied a Persian‐translated modifiable activity questionnaire (MAQ) to estimate physical activity levels (Momenan et al. 2012). The previous study reported moderate validity and high reliability of this questionnaire. The time, frequency, and intensity of light, middle, high, and challenging activities were recorded during the last year based on routine daily activities. The activity data were transformed into metabolic equivalent/min/week (Met/min/week).

## 5 Blood Pressure and Anthropometric Measurements

Weight (kg) and height (cm) were measured with a digital scale (Seca 707) (accuracy 100 g) with light clothes and no shoes in the standing position and a nonflexible tape measure (accuracy 0.5 cm), respectively. The measurement of waist circumference (WC) was performed after exhaling without pressure on the body's surface with the light clothing. This was accurate to 0.1 cm. A standardized mercury sphygmomanometer was applied to determine the systolic and diastolic blood pressure (SBP and DBP) (mmHg) according to accepted protocols, which were explained previously (Azizi et al. 2009; Hosseini‐Esfahani et al. 2018b).

## 6 Biochemical Analysis

The technician collected blood samples between 7:00 and 9:00 a.m. after 12–14 h of overnight fasting. They analyzed blood samples on the day of blood collection using the Selectra 2 auto‐analyzer at the TLGS research laboratory. They computed FBG and 2 h postprandial glucose concentrations using the enzymatic colorimetric method and the glucose oxidase technique (Vital Scientific, Spankeren, the Netherlands). In the follow‐up, all participants who did not take glucose‐lowering medications were given 82.5 g glucose monohydrate solution (equivalent to 75 g anhydrous glucose) orally for measuring 2 h postprandial glucose.

TGs and total cholesterol (TC) concentrations were estimated by an enzymatic colorimetric method using glycerol phosphate oxidase, cholesterol esterase, and cholesterol oxidase, respectively (Pars Azmoon Inc., Tehran, Iran). High‐density lipoprotein cholesterol (HDL‐C) concentration was evaluated after precipitation of the apolipoprotein B‐containing lipoproteins with phosphotungstic acid. The inter‐ and intra‐assay coefficients of variations of glucose were 2.2%. The inter‐ and intra‐assay coefficients of variations of TG were 1.6% and 0.6%, respectively.

## 7 Outcome Definition

Subjects with three or more of the following criteria were diagnosed as MetS phenotype according to the Iranian modified National Cholesterol Education Program/Adult (Grundy et al. 2004; Azizi et al. 2010): [1] Abdominal obesity (WC ≥ 95 cm in men and women); [2] BP ≥ 130/85 mmHg or antihypertensive drug treatment; [3] HDL‐C < 1.30 mmol/L (< 50 mg/dL) in women, and < 1.04 mmol/L (< 40 mg/dL) in men or receiving drug treatment; [4] FBG ≥ 6.11 mmol/L (≥ 110 mg/dL) or drug treatment for hyperglycemia; [5] TG ≥ 1.70 mmol/L (≥ 150 mg/dL) or drug treatment.

## 8 Statistical Analyses

The IBM SPSS software, version 20, was applied for data analysis. A two‐sided *p* < 0.05 was recognized as statistically significant. To compare the mean and frequency of participants' baseline characteristics across quartiles of the acrylamide intake, a *χ*2 test and one‐way ANOVA were used for categorical and continuous variables, respectively. *p* for trend across the acrylamide intake quartiles was done by designating continuous variables in a linear regression model. Multivariable Cox proportional hazard regression analyses were done to assess the hazard ratio (HR) and 95% confidence interval (CI) for MetS or its risk factors' incidence. There were no interactions between the acrylamide intake and age or sex concerning MetS incidence. The first quartile was considered as the reference. The confounders were chosen according to the literature. Also, we applied each confounder in the univariable Cox regression model; a two‐tailed *p*‐value < 0.2 was used for specifying admission in the model. The variance inflation factors were checked through regression analysis. All values in the study had scores close to 1 and less than 5.

The median of each quartile of acrylamide intake was used as a continuous variable for estimating the *p* for trend (across quartiles) in the Cox proportional hazard regression models. The definition of time to event was based on the midtime between baseline and the event date (for incidence cases) or the time between baseline and last follow‐up (for censored subjects), whichever occurred first.

Two models were constructed: the first one adjusted for age and sex, and the second one was additionally adjusted for education levels (> 14 and ≤ 14 years), physical activity (MET/min/week), energy intake (kcal/day), saturated fat intakes (percentage of energy), fiber (gr/1000 kcal), smoking (never smoked, past smoker, and current smoker), and body mass index (BMI) (kg/m^2^). In models to estimate the HR of high BP, high TG, high FBG, and low HDL‐C, respectively, the continuous amount of each risk factor at the beginning of the study corresponding to each model was added to the adjustment factors. The continuous carbohydrate intake was added to the adjustment factors in models to estimate the HR of high TG and high WC.

The proportional hazard assumption was confirmed by the Schoenfeld residuals test and plot of log [−log (survival)] vs. log (time) to see if they are parallel.

## 9 Results

In the analysis for the relationship of acrylamide intake and MetS, 4548 subjects entered, of which 1762 and 2786 were men and women, respectively. The mean ages of men and women were 38.6 ± 14.3 and 35.9 ± 11.8, respectively. The subjects consisted of 1279 incident cases of MetS with a median follow‐up of 6.23 years. There were 1002, 1391, 1373, 1009, and 1385 incident cases for low HDL‐C, high FBG, high TG, high WC, and high BP, respectively.

Table 1 shows the baseline attribute of subjects beyond quartiles of the acrylamide intake. Subjects in the higher quartiles of acrylamide intake were younger than subjects in the lower quartiles. There was a significant association between sex and acrylamide intake quartiles. The percentage of current smokers was higher in the top quartile of acrylamide intake than in the lowest quartile. Also, individuals in the higher quartiles of acrylamide intake had higher physical activity and education levels than those in the lower quartiles. There were no associations between BMI, SBP, DBP, TG, and FBG across quartiles of acrylamide intake. Subjects in the higher quartiles of acrylamide intake had lower HDL‐C (*p* < 0.001) and higher WC (*p* = 0.003) in comparison to lower quartiles.

**TABLE 1:** Baseline characteristics of adult participants of the Tehran Lipid and Glucose Study across quartiles of acrylamide intake (*n* = 4548).

	Quartiles of acrylamide intake				
variables	Q1	Q2	Q3	Q4	*p*
Acrylamide intake (μg/day)	23.6 ± 6.0	39.6 ± 4.3	56.7 ± 6.0	110 ± 74.2	
Baseline age (years)	39.6 ± 13.6	37.3 ± 13.0	36.0 ± 12.5	34.9 ± 11.7	< 0.001
Sex *n* (% women)	876 (77%)	735 (64.6%)	616 (54.2%)	559 (49.2%)	< 0.001
Current smoker *n* (%)	71 (6.2%)	81 (7.1%)	101 (8.8%)	176 (15.5%)	< 0.001
Physical activity (MET/min/week)	485 ± 674	520 ± 703	577 ± 850	630 ± 985	0.02
BMI (kg/m^2^)	26.0 ± 4.4	25.8 ± 4.4	25.6 ± 4.1	25.7 ± 4.2	0.12
WC (cm)	85.9 ± 11.6	86.4 ± 11.3	86.9 ± 11.5	87.7 ± 11.3	0.003
SBP (mm Hg)	108 ± 14.5	107 ± 14.0	108 ± 13.1	108 ± 12.3	0.67
DBP (mm Hg)	71.5 ± 9.6	71.9 ± 9.4	71.9 ± 9.6	72.3 ± 9.3	0.27
Total cholesterol (mg/dL)	182 ± 36.7	180 ± 36.2	179 ± 37.1	178 ± 35.2	0.03
TG (mg/dL)^a^	97.5 (72.9–129)	100 (73.7–133)	100 (75.2–134)	97.5 (74.4–133)	0.85
HDL‐C (mg/dL)	49.4 ± 11.5	48.4 ± 11.1	47.5 ± 10.8	47.1 ± 10.4	< 0.001
FBG (mg/dL)	89.7 ± 16.3	90.1 ± 15.4	88.6 ± 9.4	89.6 ± 13.2	0.07
Education					
Elementary	176 (15.5%)	121 (10.6%)	101 (8.9%)	79 (6.9%)	< 0.001
Diploma	693 (60.9%)	704 (61.9%)	674 (59.3%)	686 (60.3%)	
Higher diploma	268 (23.6%)	312 (27.4%)	362 (31.8%)	372 (32.7%)	

*Note:* Values are Mean ± SD unless otherwise listed. *p* values were derived from analysis of variance (ANOVA test) and Chi square test was used for continuous and dichotomous variables, respectively.

Abbreviations: BMI, body mass index; DBP, diastolic blood pressure; FBG, fasting blood glucose; SBP, systolic blood pressure; TG, triglyceride; WC, waist circumference.

^a^ Median (interquartile range).

Moreover, participants with higher acrylamide intake had significantly higher carbohydrate, total fat, and energy intake than those in lower quartiles (Table 2). Participants in the higher quartiles of acrylamide intake had significantly higher fiber intake than the lowest quartile (*p* = 0.04).

**TABLE 2:** Dietary intakes across quartiles of acrylamide intake in adult participants of the Tehran Lipid and Glucose Study (*n* = 4548).

Variables	Quartiles of acrylamide intake				*p* trend
	Q1	Q2	Q3	Q4	
Acrylamide intake (μg/day)	23.6 ± 6.0	39.6 ± 4.3	56.7 ± 6.0	110 ± 74.2	
Carbohydrate (% of energy)	57.9 ± 5.92	58.7 ± 5.32	58.9 ± 5.22	59.2 ± 5.85	0.008
Protein (% of energy)	14.6 ± 2.86	14.6 ± 2.39	14.7 ± 2.20	14.8 ± 3.39	0.05
Total fat (% of energy)	30.8 ± 5.61	29.9 ± 5.10	29.9 ± 5.0	29.7 ± 7.30	0.02
SFA (% of energy)	10.1 ± 2.40	9.84 ± 3.69	9.67 ± 2.19	9.71 ± 6.08	0.06
PUFA (% of energy)	6.26 ± 1.90	6.05 ± 1.61	6.07 ± 1.52	6.14 ± 5.84	0.90
MUFA (% of energy)	10.4 ± 2.50	10.0 ± 2.10	10.0 ± 2.16	10.1 ± 5.96	0.23
Fiber (gr/1000 kcal)	10.3 ± 3.20	10.0 ± 2.70	9.84 ± 2.67	9.69 ± 3.15	0.04
Energy intake (kcal)	1879 ± 478	2191 ± 486	2470 ± 528	2808 ± 886	< 0.001

*Note:* Values are Mean ± SD. ANOVA test; *p* for trend across percentile acrylamide intake was performed by assigning continuous variables in a liner regression model.

HR and 95% CI of MetS and its risk factors incidence across quartiles of acrylamide intake are shown in Table 3. MetS and low HDL‐C incidence were positively associated with quartiles of acrylamide intake in crude models; however, these associations were not significant after adjusting for confounding factors. Acrylamide intake was not related to high FBG in crude and adjusted models. Participants in the fourth quartile of acrylamide intake had, respectively, 15% and 21% higher risk of high TG and high WC after adjusting for confounding factors. Moreover, acrylamide intake was positively related to high BP incidence in two crude and adjusted models (HR (CI) Q1‐Q4: 1, 1.0 [0.92–1.08], 1.10 [1.02–1.19], 1.16 [1.07–1.26], *p* trend = 0.003).

**TABLE 3:** HRs (95% CI) of metabolic syndrome incidence and its risk factors across quartiles of acrylamide intake in adult participants of the Tehran Lipid and Glucose Study.

Quartiles of acrylamide						
Variables		Q1	Q2	Q3	Q4	*p* trend
Median acrylamide (μg/day)		24.5	39.6	56.1	89.6	
Case/event MetS		1137/323	1137/296	1137/330	1137/330	
HRs (95% CI) MetS	Crude	Ref	0.97 (0.89–1.06)	1.05 (0.96–1.14)	1.09 (1.0–1.19)	0.01
	Adjusted model	Ref	0.95 (0.87–1.03)	1.01 (0.93–1.10)	1.02 (0.93–1.11)	0.36
Case/event low HDL‐C		696/263	696/245	696/235	696/259	
Median acrylamide(μg/day)		20.3	35.2	53.6	92.1	
HRs (95% CI) low HDL‐C	Crude	Ref	0.99 (0.89–1.10)	1.03 (0.92–1.15)	1.14 (1.02–1.27)	0.01
	Adjusted model	Ref	0.99 (0.89–1.11)	1.03 (0.92–1.15)	1.11 (0.98–1.25)	0.07
Case/event high FBG		1303/356	1304/348	1304/357	1303/330	
Median acrylamide(μg/day)		24.7	39.8	56.4	89.2	
HRs (95% CI) high FBG	Crude	Ref	0.97 (0.98–1.04)	1.05 (0.97–1.14)	1.02 (0.94–1.10)	0.22
	Adjusted model	Ref	0.94 (0.87–1.02)	1.04 (0.96–1.13)	0.99 (0.91–1.07)	0.59
Case/event high TG		1053/350	1054/331	1054/344	1055/348	
Median acrylamide(μg/day)		19.8	33.7	51.4	90.0	
HRs (95% CI) high TG	Crude	Ref	1.05 (0.97–1.15)	1.10 (1.01–1.20)	1.15 (1.06–1.26)	< 0.001
	Adjusted model	Ref	1.05 (0.96–1.14)	1.09 (1.01–1.20)	1.15 (1.05–1.25)	0.001
Case/event high WC		975/248	976/245	977/253	976/263	
Median acrylamide(μg/day)		19.7	33.2	50.8	87.6	
HRs (95% CI) high WC	Crude	Ref	1.06 (0.97–1.16)	1.10 (1.0–1.20)	1.17 (1.07–1.28)	< 0.001
	Adjusted model	Ref	1.07 (0.96–1.20)	1.17 (1.04–1.32)	1.21 (1.07–1.37)	0.008
Case/event high BP		1249/372	1250/345	1250/337	1248/331	
Median acrylamide(μg/day)		20.1	33.8	51.9	89.4	
HRs (95% CI) high BP	Crude	Ref	1.02 (0.94–1.10)	1.10 (1.01–1.19)	1.17 (1.08–1.27)	< 0.001
	Adjusted model	Ref	1.0 (0.92–1.08)	1.10 (1.02–1.19)	1.16 (1.07–1.26)	0.003

*Note:* The median of each quartile of acrylamide intake was used as a continuous variable for estimating the P for trend in the Cox proportional hazard regression models. Crude models adjusted for age, sex; the second models were additionally adjusted for education levels, physical activity, total energy and saturated fat intakes, BMI and smoking. In models to estimate the HR of high BP, high TG, high FBG, and low HDL‐C respectively, the continuous amount of each risk factor at the beginning of study corresponding to each model was added to the adjustment factors. In models to estimate the HR of high TG, high WC the continuous amount of carbohydrate intake was added to the adjustment factors.

Abbreviations: BMI, body mass index; DBP, diastolic blood pressure; FBG, fasting blood glucose; SBP, systolic blood pressure; TG, triglyceride; WC, waist circumference.

## 10 Discussion

The associations between acrylamide intake and the incidence of MetS and its risk factors were explored in the current prospective cohort study with a follow‐up of 6.23 years. This study found no apparent association between acrylamide intake and MetS incidence in the adjusted model; however, among the MetS risk factors, significant positive relationships between acrylamide intake and the risk of hypertriglyceridemia, central obesity, and high BP were obtained after adjusting for multiple confounding factors.

As far as the literature shows, this is the first study to highlight the association between dietary acrylamide intake and the risk of MetS incidence; previous studies have evaluated the metabolic effects of biomarkers of internal exposure to acrylamide, measured as adducts of acrylamide and glycidamide hemoglobin (HbAA and HbGA) (Hung et al. 2021; Wan et al. 2022). Consistent with recent study, no significant relationship was observed between acrylamide biomarkers and the risk of MetS in the Hung et al. (2021) study; also, they reported a significant negative association between HbAA and high TG and low HDL‐C and between HbGA and high FBG. Urinary acrylamide metabolite had linear positive and dose response association with FBG due to oxidative DNA damage and lipid peroxidation (Wang et al. 2020). In the other research by (Cheang et al. 2021) high acrylamide hemoglobin biomarkers, including HbGA and HbGA/HbAA, had positive linear association with TG and negative correlation with HDL‐C. The inverse association between HbAA and hypertension, high FBG, abdominal obesity, hypertriglyceridemia, and low HDL‐C and the direct association between HbGA/HbAA and hypertriglyceridemia, low HDL‐C and abdominal obesity were the main results of Wan et al. (2022) study. Long‐term acrylamide exposure induces pro‐inflammatory state and lipid peroxidation (Kim et al. 2015). Also acrylamide exposure induces liver damage due to various oxidative pathways that may contribute to lipid changes (Watzek et al. 2012).

There were positive association between HbGA and the ratio of HbAA/HbGA with obesity, abdominal obesity, and overweight along with the negative association between HbAA and obesity in the (Huang et al. 2018) study, which might be due to disturbance in metabolic homeostasis. These results were consistent with the findings of recent study. In rats, acrylamide enhances the adipocyte differentiation through increased expression of obesity‐promoting genes (Lee and Pyo 2019).

In the studies mentioned above, the relationship between the acrylamide biomarkers and MetS risk factors were determined, while in recent study, the association between long‐term consumption of dietary acrylamide and MetS has been considered. Although the use of hemoglobin acrylamide biomarkers is a more objective method than estimating dietary intake using FFQ in recent study (Kütting et al. 2008), these biomarkers reflect the amount of acrylamide in the body just over the life span of erythrocytes, which is 4 months (Pedersen et al. 2022). In contrast, in recent study, the acrylamide intake has been monitored for more than 6 years. In addition, due to sustained endogenous acrylamide formation in the human body (Goempel et al. 2017), it is not known whether acrylamide that reacts with hemoglobin is due to dietary intake or due to internal production. Also, it is not known whether acrylamide produced in the body can induce metabolic effects or not (Huang et al. 2018). Certainly, answering these questions requires more studies.

In recent study, a higher acrylamide intake was associated with an increased risk of hypertriglyceridemia. More than 50% of acrylamide intake in the study population is driven by consuming carbohydrate foods cooked at temperatures more than 120°C (Hosseini‐Esfahani et al. 2023). Moreover, consuming high acrylamide leads to oxidative destruction of the liver (Bo et al. 2020) and changing the balance of sex hormones, which may lead to dyslipidemia (Chu et al. 2020). Consuming more carbohydrates can harm lipid profiles (Mensink et al. 2003); however, carbohydrate intake was adjusted in the analysis of this study, so the positive relationship between acrylamide intake and the incidence of high TG and central obesity is independent of carbohydrate intake.

The metabolism of acrylamide leads to the depletion of antioxidant enzymes, including glutathione, and thereby increases oxidative stress and inflammation in the body (Amirshahrokhi 2021). This chronic inflammation can eventually disrupt glucose homeostasis by disrupting the insulin signal (Tsalamandris et al. 2019). On the other hand, this oxidative stress can disrupt nitric oxide production and thereby increase BP (Lubos et al. 2008). Imbalance of sex hormones (Chu et al. 2020), changes in intestinal flora (Wang et al. 2021), and disruption of the thyroid gland (Khan et al. 1999) due to the consumption of more acrylamide can be considered as mechanisms explaining the positive relationship between acrylamide intake and the occurrence of abdominal obesity.

This study had limitations to be considered, including not evaluating passive smoker participants, while this variable can change the amount of acrylamide exposure in our study. Failure to measure the amount of excreted acrylamide is another limitation of this study; however, this research is the first study on the relationship between acrylamide intake and the risk of MetS. It can be a basis for future studies. The prospective design and high sample size of this study are considered the most important strengths. Many confounding factors have been adjusted in this study to eliminate the effects of bias for the study results.

## 11 Conclusion

During a follow‐up of 6.23 years, dietary acrylamide intake was not related to MetS, low HDL‐C, and high FBG incidence after adjusting for confounding factors.

Long‐term dietary acrylamide intake was associated with an increased risk of MetS risk factors, including hypertriglyceridemia, high BP, and abdominal obesity.

## Author Contributions

**Firoozeh Hosseini‐Esfahani:** conceptualization (lead), formal analysis (lead), investigation (lead), methodology (lead), software (supporting), supervision (supporting), writing – original draft (lead), writing – review and editing (lead). **Azam Ildarabadi:** data curation (supporting), formal analysis (supporting), investigation (supporting), methodology (supporting), software (supporting), writing – original draft (equal), writing – review and editing (equal). **Shahrzad Daei:** data curation (equal), formal analysis (equal), investigation (equal), methodology (supporting), writing – original draft (equal), writing – review and editing (equal). **Glareh Koochakpoor:** data curation (equal), investigation (equal), writing – original draft (equal), writing – review and editing (equal). **Amene Nematollahi:** data curation (equal), writing – original draft (equal), writing – review and editing (equal). **Parvin Mirmiran:** conceptualization (supporting), data curation (supporting), funding acquisition (lead), investigation (supporting), methodology (supporting), project administration (lead), resources (supporting), software (supporting), supervision (supporting), visualization (supporting), writing – original draft (supporting), writing – review and editing (supporting). **Fereidoun Azizi:** conceptualization (supporting), data curation (supporting), investigation (supporting), methodology (supporting), project administration (lead), resources (lead), supervision (lead), validation (lead), visualization (lead), writing – original draft (supporting), writing – review and editing (supporting).

## Ethics Statement

This study was approved by Nutrition and Endocrine Research Center, Research Institute for Endocrine Sciences, Shahid Beheshti University of Medical Sciences, Tehran, Iran.

## Consent

All participants filled and signed an informed consent form to participate in the study.

## Conflicts of Interest

The authors declare no conflicts of interest.

## Data Availability

The data that support the findings of this study are available on request from the corresponding author. The data are not publicly available due to privacy or ethical restrictions.
